# Cognitive function in generally healthy adults age 70 years and older in the 5-country DO-HEALTH study: MMSE and MoCA scores by sex, education and country

**DOI:** 10.1007/s40520-025-02946-4

**Published:** 2025-03-17

**Authors:** Melanie Kistler-Fischbacher, Ghazala Gohar, Caroline de Godoi Rezende Costa Molino, Katharina Geiling, Tatjana Meyer-Heim, Reto W. Kressig, E. John Orav, Bruno Vellas, Sophie Guyonnet, José A.P. da Sliva, René Rizzoli, Gabriele Armbrecht, Elisabeth Steinhagen-Thiessen, Andreas Egli, Heike A. Bischoff-Ferrari

**Affiliations:** 1 Centre on Aging and Mobility, University of Zurich, Zurich, Switzerland; 2 Department of Geriatric Medicine and Aging Research, University of Zurich, Zurich, Switzerland; 3 City of Zurich Geriatrics Department, Senior Health Centers of the City of Zurich, Zurich, Switzerland; 4 University Clinic for Aging Medicine, City Hospital Zurich, Zurich, Switzerland; 5 University Department of Geriatric Medicine FELIX PLATTER, University of Basel, Basel, Switzerland; 6 Department of Biostatistics, Harvard T.H. Chan School of Public Health, Boston, MA USA; 7 IHU HealthAge, University Hospital Toulouse and University of Toulouse III, Toulouse, France; 8 Centro Hospitalare Universitário de Coimbra, Coimbra, Portugal; 9 Centre for Innovative Biomedicine and Biotechnology (CIBB), Faculty of Medicine, University of Coimbra, Coimbra, Portugal; 10 Division of Bone Diseases, Faculty of Medicine, Geneva University Hospitals, Geneva, Switzerland; 11 Department of Radiology, Charité-Universitätsmedizin Berlin, Corporate Member of Freie Universität Berlin and Humboldt-Universität Zu Berlin, Berlin, Germany; 12 FRIEDE SPRINGER – Cardiovascular Prevention Center, Charité Universitätsmedizin, Campus Benjamin Franklin, Berlin, Germany

**Keywords:** Cognitive function, Montreal cognitive assessment, Mini mental state examination, Observational study

## Abstract

**Background:**

Mini Mental State Examination (MMSE) and Montreal Cognitive Assessment (MoCA) are validated and frequently used screening tools for cognitive function.

**Aims:**

To present MMSE and MoCA scores by sex, age and education among community-dwelling older adults.

**Methods:**

This is a post-hoc observational analysis using data from the DO-HEALTH trial, which included generally healthy adults (≥ 70 years) from Switzerland, Germany, Austria, France, and Portugal who scored at least 24 points on the MMSE at baseline. We present MMSE and MoCA scores overall and by country, sex, age (70–74 years, ≥ 75 years), education (≤ and > median education years).

**Results:**

2151 DO-HEALTH participants (mean age 74.9 years, 57% aged 70–74 years, 62% women) were included. The median (IQR) years of education was 12 (10–15), median MMSE score was 29 (28–30) and median MoCA score was 26 (23–28) points. In subgroups by sex, age, and education, the median MMSE score remained 29 for all subgroups, except for participants with shorter education (≤ 12 years) and higher age (≥ 75), who scored 28 points. For MoCA, the median score in subgroups ranged from 24 to 27 points. Participants with shorter education (≤ 12 years) and higher age (≥ 75) had lowest scores (men 24, women 25 points).

**Conclusions:**

We provide MMSE and MoCA scores for generally healthy, community-dwelling older adults from Switzerland, Germany, Austria, France and Portugal. The median MMSE and MoCA scores differed with age and education, and – less consistently – with sex.

**Trial registration:**

International Trials Registry (clinicaltrials.gov; registration ID: NCT01745263), registered December 2012.

**Supplementary Information:**

The online version contains supplementary material available at 10.1007/s40520-025-02946-4.

## Introduction

With a projected doubling of adults aged 60 years and older from 2015 to 2050, the number of people suffering from dementia is expected to rise from 50 million (2019) to 131 million (2050) worldwide [1, 2]. Being the seventh leading cause of death globally and driver of disability at older age [3], dementia has a strong socioeconomic impact [4]. In order to address this public health challenge, early and accurate identification of cognitive decline is crucial. Reliable screening tools that are easy to administer and interpret are widely used as a first step in the process of diagnosing dementia.

The Mini Mental State Examination (MMSE) [5] and Montreal Cognitive Assessment (MoCA) [6], are the most widely used screening tools for cognitive impairment and are well validated [7, 8]. It has been suggested, that performance in these screening tests may vary based on demographic (e.g., age [9, 10], sex [11, 12]) and socioeconomic (e.g., education [12]) factors but also across different languages [13]. Existing studies indeed show considerable variation across different countries for both MMSE [11, 12, 14] and MoCA scores [10, 15–17]. Establishing reference values specific for different subgroups of sex, age and education may thus improve accuracy of these screening tests. To the best of our knowledge, such data for MMSE and/or MoCA are lacking for Switzerland, Germany, Austria and France but have been published for Portugal [18–20].

In this study, we take advantage of the DO-HEALTH trial, which included older adults aged 70 years and older from five European countries. The aim of the present study is to provide MMSE and MoCA scores overall and by country for the subgroups of sex, age and education.

## Methods

### Study design and participants

This is a post-hoc observational analysis of baseline data from the DO-HEALTH trial. DO-HEALTH is a multicentre, double-blind, placebo-controlled, randomized controlled clinical trial, which was conducted in 2157 community-dwelling older adults from five European countries: Switzerland (*n* = 1006), Austria (*n* = 200), Germany (*n* = 350), France (*n* = 300), and Portugal (*n* = 301). The primary aim of DO-HEALTH was to examine the effect of the vitamin D3, omega-3 fatty acids (omega-3s), and a simple home exercise program (SHEP) on six primary endpoints: systolic and diastolic blood pressure, Short Physical Performance Battery (SPPB), MoCA, incident non-vertebral fractures and incident infections over three years [21, 22]. Eligibility criteria included age ≥ 70 years, MMSE score ≥ 24, living in the community and no major health events (e.g., myocardial infarction) in the five years prior to enrolment. The present study includes all participants with MoCA scores and education data at baseline. The trial protocol, including a full list of eligibility criteria, has been published [21]. Participants were recruited between December 2012 and November 2014. The protocol was approved by ethics and regulatory agencies of all five countries [21] and registered in the International Trials Registry (clinicaltrials.gov; registration ID: NCT01745263). All participants provided informed consent in their national languages.

### Assessment of cognitive function

Cognitive function was assessed by the MMSE and MoCA. The MMSE is an 11-item assessment that examines visuospatial abilities, language, concentration, working memory, memory recall and orientation. The MMSE score ranges from 0 to 30 points, with higher values indicating better cognitive function [5]. It is validated for community-dwelling cognitively healthy older adults [23]. MoCA covers visuospatial abilities, short-term memory recall, abstract reasoning, attention/concentration, language, orientation and executive functions. It’s score ranges from 0 to 30, with higher values indicating better cognitive function [6]. It is validated for community-dwelling older adults [24]. Both tests take approximately 10 min to complete.

### Other measures

Sociodemographic and anthropometric information included age, sex, body mass index (BMI), education and country [22]. Education was assessed as the number of years of formal education completed. For the purpose of this manuscript, the term “shorter” education is used if the years of educations was equal or lower than the country’s median years of education, while “longer” indicates more years of education than the median. For age, “younger” refers to age 70–74 years and “older” to age 75 years and older. The number of comorbidities was assessed with the Self-Administrated Comorbidity Questionnaire [25], which assesses the presence of 12 common chronic conditions. Mental health was assessed with the 15-item Geriatric depression scale (GDS) [26], a widely used instrument developed for geriatric populations [27]. The maximum score is 15 with lower scores indicating lower likelihood of depression. Self-rated health was assessed with the EuroQoL-visual analogue scale (EQ-VAS) [28]. Participants responded to the question “I would like you to tell me the point on the scale where you would place your current state of health” by marking a number on a visual scale that reflected their perceived health. The scale ranges from 0 (‘very poor’ health) to 100 (‘very good’ health).

All data were collected by trained study nurses and medical doctors using standardized questionnaires and assessments, based on standard operating procedures. All participants were evaluated in their national languages.

### Statistical analysis

MoCA and MMSE scores are presented overall and for each country by the subgroups of sex (men, women), age (70–74 years, ≥ 75 years) and education. For education, we used the median years of the specific country as a cutoff in the primary analysis (≤ median and > median years of education). The reason for this approach is the differing educational systems across countries and the known influence of education on cognitive performance [6, 20]. In a sensitivity analysis, we used the median of the overall sample as a cutoff to verify similarities between countries among participants with higher education. MoCA scores were not adjusted for education as we present it by median years of education. Scores are presented as median and interquartile range (IQR; 25th and 75th percentile) in the main text of the manuscript. Additional percentiles (1st, 5th, 10th, 90th, 95th, 99th) as well as means and standard deviations (SD) are presented in the supplemental material. Statistical analysis were performed using SAS version 9.4 (SAS Institute, Inc., Cary, NC, United States).

## Results

### Baseline characteristics

We included 2151 out of 2157 DO-HEALTH participants since MoCA scores were missing for 6 participants. Participant characteristics are shown in Table 1. Overall, the mean age was 74.9 (SD 4.4) years, 61.7% were women and the median years of education was 12 (IQR 10–15). Participants were generally healthy with a median number of comorbidities of 2 and a median GDS-15 score of 1.

**Table 1:** Baseline characteristics of study participants^a^

	Overall *n* = 2151	Switzerland *n* = 1004	Germany *n* = 350	Austria *n* = 199	France *n* = 297	Portugal *n* = 301
Age [years], mean (SD)	74.9 (4.4)	75.2 (4.6)	73.3 (2.7)	74.1 (4.1)	75.5 (4.6)	76.0 (5.0)
Age 70–74 years, n (%) Age ≥ 75 years, n (%)	1234 (57.4) 917 (42.6)	540 (53.8) 464 (46.2)	268 (76.6) 82 (23.4)	139 (69.9) 60 (30.2)	139 (46.8) 158 (53.2)	148 (49.2) 153 (50.8)
Women, n (%)	1327 (61.7)	607 (60.5)	247 (70.6)	102 (51.3)	179 (60.3)	192 (63.8)
MMSE, median (IQR) ^b^	29 (28–30)	29 (28–30)	29 (28–30)	29 (28–30)	29 (27–29)	27 (26–29)
MoCA, median (IQR) ^c^	26 (23–28)	26 (24–28)	25 (23–27)	27 (25–28)	27 (25–28)	21 (18–25)
BMI [kg/m^2^], mean (SD) ^d^	26.3 (4.3)	25.8 (4.2)	26.8 (4.3)	25.2 (3.9)	25.8 (4.2)	28.7 (4.1)
Years of education, mean (SD) ^e^	12.6 (4.3)	13.4 (3.4)	14.5 (3.3)	12.0 (3.7)	13.3 (3.9)	7.9 (5.3)
Years of education, median (IQR)	12 (10–15)	13 (11–15)	14 (12–17)	11 (10–13)	14 (11–16)	6 (4–12)
Education ≤ 12 years, n (%)	1105 (51.34)	485 (48.3)	106 (30.3)	144 (72.7)	132 (44.4)	238 (79.1)
Education > 12 years, n (%)	1046 (48.6)	519 (51.7)	244 (69.7)	55 (27.6)	165 (55.6)	63 (20.9)
Number of comorbidities, median (IQR) ^f^	2 (1–3)	1 (0–8)	2 (0–7)	1 (0–6)	2 (0–7)	2 (0–8)
GDS-15 Score, median (IQR) ^g^	1 (0–2)	1 (0–2)	1 (0–8)	1 (0–8)	2 (0–13)	3 (0–14)
EQ-VAS Score, median (IQR) ^h^	82 (1–100)	88 (1–100)	81 (1–100)	90 (32–100)	80 (22–100)	78.00 (2–100)

Abbreviations: BMI, body mass index; EQ-VAS, EuroQoL-visual analogue scale; GDS-15, Geriatric depression scale 15; MMSE, Mini Mental State Examination; MoCA, Montreal Cognitive Assessment; IQR, Interquartile range; SD, standard deviation

^a^ Medians and IQRs are presented for non-normally distributed variables

^b^ The MMSE has a range of 0 to 30 points. Scores greater than 24 suggest normal cognitive function

^c^ The MoCA has a range of 0 to 30 points. Scores greater than 26 suggest normal cognitive function

^d^ Body mass index (BMI) was calculated as weight in kilograms by height in meters squared. Higher BMI values reflect overweight (≥ 25) and obesity (≥ 30)

^e^ Years of education is normally distributed and therefore is reported as mean (SD). Additionally, years of education is presented as median (IQR) in order to report the country-specific median

^f^ Comorbidity was measured by the Self-Administered Comorbidity Questionnaire which assesses the presence of 12 common chronic conditions

^g^ The GDS-15 score ranges from 0 to 15 and lower scores are better

^h^ The EQ-VAS score ranges from 0 to 100 and higher scores are better

Overall, the median MMSE score was 29 (IQR 28–30). The median MMSE scores ranged from 27 (26–29) in Portugal to 29 (28–30) in Switzerland, Germany and Austria. The median MoCA scores ranged from 21 (18–25) in Portugal to 27 (25–28) in Austria and France.

### MMSE scores in subgroups of sex, age and country-specific education

#### Overall

In subgroups by sex, age, and education, the median MMSE score remained 29 for all subgroups, except for participants (both men and women) with shorter education (≤ 12 years) and higher age (≥ 75 years), who scored 28 points (Table 2; Supplemental Table 1).

**Table 2:** MMSE scores by sex, age and education, overall and by country

Overall (*N* = 2151)				
	Women		Men	
	70–74 years	≥ 75 years	70–74 years	≥ 75 years
Education ≤ 12 years	29 (27–30) [*n* = 413]	28 (27–29) [*n* = 375]	29 (27–30) [*n* = 163]	28 (27–29) [*n* = 154]
Education > 12 years	29 (28–30) [*n* = 354]	29 (28–30) [*n* = 185]	29 (28–30) [*n* = 304]	29 (28–29) [*n* = 203]
**Switzerland** (*n*** = 1004)**				
	Women		Men	
	70–74 years	≥ 75 years	70–74 years	≥ 75 years
Education ≤ 13 years	29 (28–30) [*n* = 217]	29 (28–30) [*n* = 223]	29 (28–30) [*n* = 88]	29 (28–30) [*n* = 91]
Education > 13 years	29 (29–30) [*n* = 108]	30 (29–30) [*n* = 59]	29 (28–30) [*n* = 127]	29 (28–30) [*n* = 91]
**Germany** (*n*** = 350)**				
	Women		Men	
	70–74 years	≥ 75 years	70–74 years	≥ 75 years
Education ≤ 14 years	29 (28–30) [*n* = 115]	29 (28.5–30) [*n* = 32]	28 (27–30) [*n* = 27]	29 (27–29) [*n* = 7]
Education > 14 years	29 (28–30) [*n* = 79]	29 (29–30) [*n* = 21]	29 (28–30) [*n* = 47]	29 (28–29) [*n* = 22]
**Austria** (*n*** = 199)**				
	Women		Men	
	70–74 years	≥ 75 years	70–74 years	≥ 75 years
Education ≤ 11 years	29 (28–30) [*n* = 44]	29 (28–29) [*n* = 18]	28.5 (27–29) [*n* = 26]	29 (28–30) [*n* = 14]
Education > 11 years	30 (29–30) [*n* = 29]	28 (28–30) [*n* = 11]	29 (28–30) [*n* = 40]	29 (28–29) [*n* = 17]
**France** (*n*** = 297)**				
	Women		Men	
	70–74 years	≥ 75 years	70–74 years	≥ 75 years
Education ≤ 14 years	28 (27–29) [*n* = 51]	28 (27–29) [*n* = 74]	28.5 (27-29.5) [*n* = 28]	28 (27–29) [*n* = 32]
Education > 14 years	29 (28–30) [*n* = 27]	29 (28–29) [*n* = 27]	29 (28–29) [*n* = 33]	29 (28–29) [*n* = 25]
**Portugal** (*n*** = 301)**				
	Women		Men	
	70–74 years	≥ 75 years	70–74 years	≥ 75 years
Education ≤ 6 years	26 (25–27) [*n* = 57]	26 (24–27) [*n* = 59]	28 (26–29) [*n* = 17]	28 (26–29) [*n* = 29]
Education > 6 years	28 (27–30) [*n* = 40]	28 (26–29) [*n* = 36]	29 (27–30) [*n* = 34]	28 (27–29) [*n* = 29]

#### By country

For Switzerland the range of median MMSE scores was 29–30 points, for Germany 28–29 points, for Austria 28–30 points, for France 28–29 points and for Portugal 26–29 points (Table 2; Supplemental Tables 2–6).

### MoCA scores in subgroups of sex, age and country-specific education

#### Overall

For MoCA scores, we observed similar findings as for MMSE, however, with a greater range in median scores across different subgroups (24 to 27 points). Participants with shorter education (≤ 12 years) and higher age (≥ 75 years) scored lowest (men 24 points, women 25 points; Table 3; Supplemental Table 1).

**Table 3:** MoCA scores by sex, age and education, overall and by country

Overall (*N* = 2151)				
	Women		Men	
	70–74 years	≥ 75 years	70–74 years	≥ 75 years
Education ≤ 12 years	26 (23–28) [*n* = 413]	25 (22–27) [*n* = 375]	25 (23–26) [*n* = 163]	24 (21–26) [*n* = 154]
Education > 12 years	27 (25–28) [*n* = 354]	26 (24–28) [*n* = 185]	27 (24–28) [*n* = 304]	26 (24–28) [*n* = 203]
**Switzerland** (*n*** = 1004)**				
	Women		Men	
	70–74 years	≥ 75 years	70–74 years	≥ 75 years
Education ≤ 13 years	27 (24–28) [*n* = 217]	25 (23–27) [*n* = 223]	25.5 (23–27) [*n* = 88]	25 (23–27) [*n* = 91]
Education > 13 years	28 (26–29) [*n* = 108]	27 (24–29) [*n* = 59]	27 (25–29) [*n* = 127]	26 (24–28) [*n* = 91]
**Germany** (*n*** = 350)**				
	Women		Men	
	70–74 years	≥ 75 years	70–74 years	≥ 75 years
Education ≤ 14 years	25 (23–27) [*n* = 115]	26 (23–27) [*n* = 32]	25 (21–26) [*n* = 27]	22 (21–25) [*n* = 7]
Education > 14 years	26 (24–28) [*n* = 79]	26 (24–28) [*n* = 21]	25 (23–27) [*n* = 47]	24 (22–25) [*n* = 22]
**Austria** (*n*** = 199)**				
	Women		Men	
	70–74 years	≥ 75 years	70–74 years	≥ 75 years
Education ≤ 11 years	27 (25-28.5) [*n* = 44]	25.5 (23–28) [*n* = 18]	25 (22–27) [*n* = 26]	26 (24–27) [*n* = 14]
Education > 11 years	28 (25–30) [*n* = 29]	26 (24–27) [*n* = 11]	27 (25-28.5) [*n* = 40]	27 (25–27) [*n* = 17]
**France** (*n*** = 297)**				
	Women		Men	
	70–74 years	≥ 75 years	70–74 years	≥ 75 years
Education ≤ 14 years	27 (24–29) [*n* = 51]	26 (24–28) [*n* = 74]	26 (25–27) [*n* = 28]	26 (24–28) [*n* = 32]
Education > 14 years	28 (26–29) [*n* = 27]	27 (26–29) [*n* = 27]	28 (27–29) [*n* = 33]	27 (26–28) [*n* = 25]
**Portugal** (*n*** = 301)**				
	Women		Men	
	70–74 years	≥ 75 years	70–74 years	≥ 75 years
Education ≤ 6 years	20 (17–22) [*n* = 57]	17 (14–21) [*n* = 59]	23 (19–24) [*n* = 17]	20 (17–22) [*n* = 29]
Education > 6 years	25 (22–27) [*n* = 40]	23 (21–26) [*n* = 36]	24.5 (22–26) [*n* = 34]	24 (20–26) [*n* = 29]

#### By country

For the median MoCA scores by country, there was a wider range. For Switzerland the range of median MoCA scores was 25–28 points, for Germany 24–26 points, for Austria 25–28 points, for France 26–28 points and for Portugal 17–25 points (Table 3; Supplemental Tables 2–6).

### MMSE and MoCA scores in subgroups of country, sex, age and education, using the overall median years of education as a cutoff

If a consistent cutoff of 12 years was used for each country, median MMSE scores range was 29–30 points for Switzerland, 28–29 points for Germany, 28–30 points for Austria, 27.5–29 points for France and 27–29 points for Portugal.

Median MoCA scores range was 25–28 points for Switzerland, 22–26 points for Germany, 25.5–29 points for Austria, 25–28 points for France and 22–26 points for Portugal (Supplemental Tables 7–11).

## Discussion

This study provides MMSE and MoCA by sex, age and education, based on a sample of 2151 generally healthy, community-dwelling adults aged ≥ 70 years from five European countries. For MMSE, the overall median was 29 (IQR 28–30) points and there was a narrow range across the different subgroups (median range 28–29 points). For MoCA, the overall median was 26 (IQR 23–28) points and there was a wider range across subgroups (24–27 points). For both MMSE and MoCA, lowest scores were observed in older individuals with shorter education.

The overall median MMSE of 29 points (out of 30) was high, but comparable to findings from large (> 1000 participants) European cohort studies among healthy community-dwelling older adults with a similar mean age and education level [12, 15, 29]. Specifically, for Germany, our results are in line with a previous study, which only included subgroups by age and education (*N* = 1090, median MMSE score in most subgroups: 28 points) [29]. For Switzerland, Austria and France, this is the first study to publish median MMSE scores, but the median scores by subgroups mirror the overall findings. For Portugal, median MMSE scores were somewhat lower, if a country-specific education cutoff was applied. DO-HEALTH participants from Portugal had a lower level of education (median: 6 years) compared to the other four countries (11–14 years). Neither of the two existing studies from Portugal present MMSE scores by subgroups of age, sex and education [18, 30]. Furthermore, the mean number of years of education in those two studies (6.5 years [30], 7.9 years [18]) were comparable to our sample (7.9 years). If the common cutoff of 12 years of education was applied for each country, median MMSE scores for Portugal were similar to the other countries, which is in line with a Portuguese study that demonstrated significant variation in MMSE scores based on level of education (*β* = 0.485, *p* < 0.001) [18].

For median MoCA scores, we observed a wider range across the subgroups, compared to MMSE. This observation is in line with previous studies, including the original validation study of the MoCA test [6]. In fact, the ceiling effect and poor sensitivity to detect mild cognitive impairment (MCI) are known weaknesses of MMSE [6, 31]. Furthermore, a score of ≥ 24 points on MMSE was an inclusion criteria of DO-HEALTH, hence MMSE scores may have been higher. The overall median MoCA score of 26 is comparable with findings from several European cohort studies among healthy community-dwelling older adults with a similar mean age and education level [10, 32–34]. Specifically, for Switzerland, our study confirms previously published values (*N* = 283, mean age 73.8 years, median MoCA score: 26) but in a larger sample and including participants from three different Swiss cities [33]. For Portugal, a previous study did not present results by sex and used four different educational levels instead of only two, hence results are difficult to compare [20]. And, to the best of our knowledge, MoCA scores have not been published for Germany, Austria and France, previously.

Consistent with previous studies [9, 11, 12, 14, 15, 18, 35–37], MMSE and MoCA scores were found to differ by age and education. Specifically, participants with higher age and shorter education had lower median MMSE and MoCA scores. Age can affect cognition through various pathways (e.g., structural, vascular, inflammatory) and is known to be the greatest risk factor for cognitive decline [38]. Education on the other has a protective effect against cognitive decline, possibly by modulating neural networks to be more efficient and less susceptible to disruption. Furthermore, highly educated people are able to maintain a higher level of cognitive function even when pathological changes have already occurred, which may delay the onset of cognitive impairment [39]. This concept is also well known as “cognitive reserve”. Age and education are thus important factors to be considered when interpreting MMSE and MoCA scores.

The impact of sex on cognitive performance on MMSE and MoCA is less clear. For most countries, median MoCA scores were somewhat higher in women, except for Portugal, where men scored higher in each of the subgroups by age and education. It is well known that the influence of sex on cognition and risk of cognitive decline is complex. Women have a better memory and processing speed, an advantage that may extend to early stages of cognitive decline [40]. Differences in cognitive performance for men and women across different geographical regions have previously been described, but reasons for this observations are unclear [41]. With respect to published MMSE and MoCA scores, some previous studies found an influence of sex [32, 33, 42], while others did not [15, 19, 43].

This is the first study to provide MMSE and MoCA scores for five European countries, for many of which such data has not been published to date. Nevertheless, some limitations should be acknowledged. This was not a population-based study. We used baseline data from DO-HEALTH, a randomized controlled trial, which targeted generally healthy participants. Furthermore, the sample size for some subgroups by country are relatively small which should be considered when interpreting our findings.

In conclusion, this observational study provides MMSE and MoCA scores for generally healthy, community-dwelling older adults from Switzerland, Germany, Austria, France and Portugal. The performance on MMSE and MoCA differed with age and education, and - less consistently - with sex.

## Electronic supplementary material

Below is the link to the electronic supplementary material.

Supplementary Material 1

## Data Availability

No datasets were generated or analysed during the current study.

## References

[1] Organisation WH Ageing and health. [Fact Sheet] 2021 [updated 4 October 2021. Available from: https://www.who.int/news-room/fact-sheets/detail/ageing-and-health

[2] (ADI) AsDI World Alzheimer Report 2016 2016 [Available from: https://www.alzint.org/u/WorldAlzheimerReport2016.pdf

[3] Lisko I, Kulmala J, Annetorp M, Ngandu T, Mangialasche F, Kivipelto M (2021) How can dementia and disability be prevented in older adults: where are we today and where are we going? J Intern Med 289(6):807–830. 10.1111/joim.13227 33314384 10.1111/joim.13227 PMC8248434

[4] International AD, University MG Adi - World Alzheimer Report 2021. Alzheimer’s Disease International (ADI) 2021 [Available from: https://www.alzint.org/resource/world-alzheimer-report-2021/

[5] Folstein MF, Folstein SE, McHugh PR (1975) Mini-mental state. A practical method for grading the cognitive state of patients for the clinician. J Psychiatr Res 12(3):189–198. 10.1016/0022-3956(75)90026-6 1202204 10.1016/0022-3956(75)90026-6

[6] Nasreddine ZS, Phillips NA, Bédirian V, Charbonneau S, Whitehead V, Collin I et al (2005) The Montreal Cognitive Assessment, MoCA: a brief screening tool for mild cognitive impairment. J Am Geriatr Soc 53(4):695–699. 10.1111/j.1532-5415.2005.53221.x 15817019 10.1111/j.1532-5415.2005.53221.x

[7] Tombaugh TN, McIntyre NJ (1992) The mini-mental state examination: a comprehensive review. J Am Geriatr Soc 40(9):922–935. 10.1111/j.1532-5415.1992.tb01992.x 1512391 10.1111/j.1532-5415.1992.tb01992.x

[8] Zhang S, Qiu Q, Qian S, Lin X, Yan F, Sun L et al (2021) Determining appropriate screening tools and cutoffs for cognitive impairment in the Chinese Elderly. Front Psychiatry 12:773281. 10.3389/fpsyt.2021.773281 34925100 10.3389/fpsyt.2021.773281 PMC8674928

[9] Crum RM, Anthony JC, Bassett SS, Folstein MF (1993) Population-based norms for the Mini-mental State examination by age and educational level. JAMA 269(18):2386–2391 8479064

[10] Engedal K, Gjøra L, Benth J, Wagle J, Rønqvist TK, Selbæk G (2022) The Montreal Cognitive Assessment: normative data from a large, population-based sample of cognitive healthy older adults in Norway—The HUNT Study. J Alzheimers Dis 86(2):589–99. 10.3233/jad-215442 10.3233/JAD-215442 35094994

[11] Li H, Jia J, Yang Z (2016) Mini-mental State Examination in Elderly Chinese: a Population-based normative study. J Alzheimers Dis 53(2):487–496. 10.3233/jad-160119 27163822 10.3233/JAD-160119

[12] Huppert FA, Cabelli ST, Matthews FE (2005) Brief cognitive assessment in a UK population sample -- distributional properties and the relationship between the MMSE and an extended mental state examination. BMC Geriatr 5:7. 10.1186/1471-2318-5-7 15869717 10.1186/1471-2318-5-7 PMC1134657

[13] Roop S, Rolin S, Davis J (2023) B– 26 examining Montreal Cognitive Assessment (MoCA) performance by Primary and Testing Language. Arch Clin Neuropsychol 38(7):1390. 10.1093/arclin/acad067.232

[14] Ouvrard C, Berr C, Meillon C, Ribet C, Goldberg M, Zins M, Amieva H (2019) Norms for standard neuropsychological tests from the French CONSTANCES cohort. Eur J Neurol 26(5):786–793. 10.1111/ene.13890 30575234 10.1111/ene.13890

[15] Kenny RA, Coen RF, Frewen J, Donoghue OA, Cronin H, Savva GM (2013) Normative values of cognitive and physical function in older adults: findings from the Irish longitudinal study on Ageing. J Am Geriatr Soc 61(Suppl 2):S279–S290. 10.1111/jgs.12195 23662720 10.1111/jgs.12195

[16] Lu J, Li D, Li F, Zhou A, Wang F, Zuo X et al (2011) Montreal cognitive assessment in detecting cognitive impairment in Chinese elderly individuals: a population-based study. J Geriatr Psychiatry Neurol 24(4):184–190. 10.1177/0891988711422528 22228824 10.1177/0891988711422528

[17] Rossetti HC, Lacritz LH, Cullum CM, Weiner MF (2011) Normative data for the Montreal Cognitive Assessment (MoCA) in a population-based sample. Neurology 77(13):1272–1275. 10.1212/WNL.0b013e318230208a 21917776 10.1212/WNL.0b013e318230208a

[18] Freitas S, Simões MR, Alves L, Santana I (2015) The Relevance of Sociodemographic and Health variables on MMSE normative data. Appl Neuropsychol Adult 22(4):311–319. 10.1080/23279095.2014.926455 25531579 10.1080/23279095.2014.926455

[19] Freitas S, Simões MR, Alves L, Santana I (2011) Montreal Cognitive Assessment (MoCA): normative study for the Portuguese population. J Clin Exp Neuropsychol 33(9):989–996. 10.1080/13803395.2011.589374 22082082 10.1080/13803395.2011.589374

[20] Gonçalves J, Gerardo B, Nogueira J, Afonso RM, Freitas S Montreal Cognitive Assessment (MoCA): an update normative study for the Portuguese population. Appl Neuropsychol Adult. 2023:1–7. 10.1080/23279095.2023.2252949 10.1080/23279095.2023.2252949 37708840

[21] Bischoff-Ferrari HA, de Molino GRC, Rival C, Vellas S, Rizzoli B, Kressig R (2021) DO-HEALTH: vitamin D3 - Omega-3 - home exercise - healthy aging and longevity trial - design of a multinational clinical trial on healthy aging among European seniors. Contemp Clin Trials 100:106124. 10.1016/j.cct.2020.106124 32858228 10.1016/j.cct.2020.106124

[22] Bischoff-Ferrari HA, Vellas B, Rizzoli R, Kressig RW, da Silva JAP, Blauth M et al (2020) Effect of vitamin D supplementation, Omega-3 fatty acid supplementation, or a strength-training Exercise Program on Clinical outcomes in older adults: the DO-HEALTH Randomized Clinical Trial. JAMA 324(18):1855–1868. 10.1001/jama.2020.16909 33170239 10.1001/jama.2020.16909 PMC7656284

[23] Mitrushina M, Satz P (1991) Reliability and validity of the mini-mental state exam in neurologically intact elderly. J Clin Psychol 47(4):537–543. 10.1002/1097-4679(199107)47:4%3C537::aid-jclp2270470411%3E3.0.co;2-9 1939698 10.1002/1097-4679(199107)47:4<537::aid-jclp2270470411>3.0.co;2-9

[24] Luis CA, Keegan AP, Mullan M (2009) Cross validation of the Montreal Cognitive Assessment in community dwelling older adults residing in the Southeastern US. Int J Geriatr Psychiatry 24(2):197–201. 10.1002/gps.2101 18850670 10.1002/gps.2101

[25] Sangha O, Stucki G, Liang MH, Fossel AH, Katz JN (2003) The self-administered Comorbidity Questionnaire: a new method to assess comorbidity for clinical and health services research. Arthritis Rheum 49(2):156–163. 10.1002/art.10993 12687505 10.1002/art.10993

[26] Yesavage JA, Brink TL, Rose TL, Lum O, Huang V, Adey M, Leirer VO (1982) Development and validation of a geriatric depression screening scale: a preliminary report. J Psychiatr Res 17(1):37–49. 10.1016/0022-3956(82)90033-4 7183759 10.1016/0022-3956(82)90033-4

[27] Krishnamoorthy Y, Rajaa S, Rehman T (2020) Diagnostic accuracy of various forms of geriatric depression scale for screening of depression among older adults: systematic review and meta-analysis. Arch Gerontol Geriatr 87:104002. 10.1016/j.archger.2019.104002 31881393 10.1016/j.archger.2019.104002

[28] Buchholz I, Marten O, Janssen MF (2022) Feasibility and validity of the EQ-5D-3L in the elderly europeans: a secondary data analysis using SHARE(d) data. Qual Life Res. 10.1007/s11136-022-03158-3 35624409 10.1007/s11136-022-03158-3 PMC9546963

[29] Stein J, Luppa M, Maier W, Wagner M, Wolfsgruber S, Scherer M et al (2012) Assessing cognitive changes in the elderly: reliable change indices for the Mini-mental State Examination. Acta Psychiatr Scand 126(3):208–218. 10.1111/j.1600-0447.2012.01850.x 22375927 10.1111/j.1600-0447.2012.01850.x

[30] Morgado J, Rocha CS, Maruta C, Guerreiro M, Pavao Martins I (2009) New normative values of Mini-mental State Examination. Sinapse 9(2):10–16

[31] Trzepacz PT, Hochstetler H, Wang S, Walker B, Saykin AJ (2015) Relationship between the Montreal Cognitive Assessment and Mini-mental State examination for assessment of mild cognitive impairment in older adults. BMC Geriatr 15:107. 10.1186/s12877-015-0103-3 26346644 10.1186/s12877-015-0103-3 PMC4562190

[32] Borland E, Nägga K, Nilsson PM, Minthon L, Nilsson ED, Palmqvist S (2017) The Montreal Cognitive Assessment: normative data from a large Swedish population-based cohort. J Alzheimers Dis 59(3):893–901. 10.3233/jad-170203. The Montreal Cognitive Assessment: normative data from a large Swedish population-based cohort 10.3233/JAD-170203 PMC5545909 28697562

[33] Thomann AE, Goettel N, Monsch RJ, Berres M, Jahn T, Steiner LA, Monsch AU (2018) The Montreal Cognitive Assessment: normative data from a German-speaking cohort and comparison with international normative samples. J Alzheimers Dis 64(2):643–655. 10.3233/jad-180080 10.3233/JAD-180080 PMC6027948 29945351

[34] Bartos A, Fayette D (2018) Validation of the Czech Montreal Cognitive Assessment for mild cognitive impairment due to Alzheimer Disease and Czech norms in 1,552 Elderly persons. Dement Geriatr Cogn Disord 46(5–6):335–345. 10.1159/000494489 30513529 10.1159/000494489

[35] Mougias A, Christidi F, Kaldi M, Kerossi MI, Athanasouli P, Politis A (2020) Mini-mental state examination: Greek normative data stratified by age and education in a large sample of 925 community-dwelling healthy participants. Adv Exp Med Biol 1196:93–102. 10.1007/978-3-030-32637-1_9 10.1007/978-3-030-32637-1_9 32468310

[36] Kvitting AS, Fällman K, Wressle E, Marcusson J, Age-Normative MMSE (2019) Data for older persons aged 85 to 93 in a longitudinal Swedish cohort. J Am Geriatr Soc 67(3):534–538. 10.1111/jgs.15694 30536796 10.1111/jgs.15694 PMC6949533

[37] Carpinelli Mazzi M, Iavarone A, Russo G, Musella C, Milan G, D’Anna F et al (2020) Mini-mental state examination: new normative values on subjects in Southern Italy. Aging Clin Exp Res 32(4):699–702. 10.1007/s40520-019-01250-2 31230268 10.1007/s40520-019-01250-2

[38] Deary IJ, Corley J, Gow AJ, Harris SE, Houlihan LM, Marioni RE et al (2009) Age-associated cognitive decline. Br Med Bull 92:135–152. 10.1093/bmb/ldp033 19776035 10.1093/bmb/ldp033

[39] Lovden M, Fratiglioni L, Glymour MM, Lindenberger U, Tucker-Drob EM (2020) Education and cognitive functioning across the Life Span. Psychol Sci Public Interest 21(1):6–41. 10.1177/1529100620920576 32772803 10.1177/1529100620920576 PMC7425377

[40] LaPlume AA, McKetton L, Anderson ND, Troyer AK (2022) Sex differences and modifiable dementia risk factors synergistically influence memory over the adult lifespan. Alzheimers Dement (Amst) 14(1):e12301. 10.1002/dad2.12301 35386471 10.1002/dad2.12301 PMC8973898

[41] Mielke MM (2018) Sex and gender differences in Alzheimer’s Disease Dementia. Psychiatr Times 35(11):14–17 30820070 PMC6390276

[42] Konstantopoulos K, Vogazianos P, Doskas T (2016) Normative Data of the Montreal Cognitive Assessment in the Greek Population and Parkinsonian Dementia. Arch Clin Neuropsychol 31(3):246–253. 10.1093/arclin/acw002 26891720 10.1093/arclin/acw002

[43] Santangelo G, Siciliano M, Pedone R, Vitale C, Falco F, Bisogno R et al (2015) Normative data for the Montreal Cognitive Assessment in an Italian population sample. Neurol Sci 36(4):585–591. 10.1007/s10072-014-1995-y 25380622 10.1007/s10072-014-1995-y

