# Supplementary material for: Cognitive function in generally healthy adults age 70 years and older in the 5-country DO-HEALTH study: MMSE and MoCA scores by sex, education and country
Source: Aging Clin Exp Res. 2025 Mar 17;37(1):88. doi: 10.1007/s40520-025-02946-4 (PMC11913955; doi:10.1007/s40520-025-02946-4)
Supplement: Supplementary file 1 — Supplementary Material 1 [file 40520_2025_2946_MOESM1_ESM.pdf]

SUPPLEMENTAL MATERIAL

Cognitive function in generally healthy adults age 70 years and older in the 5-country DO-HEALTH study: MMSE and MoCA scores by sex, education and country

Melanie Kistler-Fischbacher, Ghazala Gohar, Caroline de Godoi Rezende Costa Molino, Katharina Geiling, Tatjana Meyer-Heim, Reto W. Kressig, E. John Orav, Bruno Vellas, Sophie Guyonnet, José A.P. da Sliva, René Rizzoli, Gabriele Armbrecht, Elisabeth Steinhagen-Thiessen, Egli Andreas, Heike A. Bischoff-Ferrari

Corresponding author
Bischoff-Ferrari Heike A., MD, DrPH
Dept. of Geriatric Medicine and Aging Research,
University of Zurich, Centre on Aging and Mobility, Tièchestrasse 99, 8037 Zürich, Switzerland
Email: heikea.bischoff-ferrari@uzh.ch

Supplemental Table 1. Percentiles for MMSE scores by sex, age and education, overall

**Overall (N = 2151)**				
**MMSE**	Women		Men	
	70-74 years	≥ 75 years	70-74 years	≥ 75 years
Education ≤ 12 years	n = 413	n = 375	n = 163	n = 154
99th percentile	30	30	30	30
95th percentile	30	30	30	30
90th percentile	30	30	30	30
75th percentile	30	29	30	29
50th percentile	29	28	29	28
25th percentile	27	27	27	27
10th percentile	26	25	26	25
5th percentile	25	24	25	24
1st percentile	24	24	25	24
mean (SD)	28.3 (1.6)	28.0 (1.8)	28.3 (1.5)	28.1 (1.7)
Education > 12 years	n = 354	n = 185	n = 304	n = 203
99th percentile	30	30	30	30
95th percentile	30	30	30	30
90th percentile	30	30	30	30
75th percentile	30	30	30	29
50th percentile	29	29	29	29
25th percentile	28	28	28	28
10th percentile	27	27	27	27
5th percentile	26	27	27	26
1st percentile	25	25	25	25
mean (SD)	28.9 (1.2)	28.9 (1.3)	28.8 (1.2)	28.6 (1.2)
**MoCA**	Women		Men	
	70-74 years	≥ 75 years	70-74 years	≥ 75 years
Education ≤ 12 years				
99th percentile	30	30	30	30
95th percentile	30	29	29	29
90th percentile	29	28	28	28
75th percentile	28	27	26	26
50th percentile	26	25	25	24
25th percentile	23	22	23	21
10th percentile	20	17	21	18
5th percentile	17	14	20	17
1st percentile	13	11	12	13
mean (SD)	24.9 (3.7)	23.6 (4.3)	24.4 (3.2)	23.4 (3.8)
Education > 12 years				
99th percentile	30	30	30	30
95th percentile	30	30	30	30
90th percentile	29	29	29	29
75th percentile	28	28	28	28
50th percentile	27	26	27	26
25th percentile	25	24	24	24
10th percentile	23	23	23	22
5th percentile	22	21	21	20
1st percentile	20	18	20	19
mean (SD)	26.5 (2.4)	26.0 (2.8)	26.2 (2.6)	25.7 (2.8)

Supplemental Table 2. Percentiles for MMSE and MoCA scores by sex, age and country-specific education for Switzerland

**Switzerland (N = 1004)**				
**MMSE**	Women		Men	
	70-74 years	≥ 75 years	70-74 years	≥ 75 years
Education ≤ 13 years	n = 217	n = 223	n = 88	n = 91
99th percentile	30	30	30	30
95th percentile	30	30	30	30
90th percentile	30	30	30	30
75th percentile	30	30	30	30
50th percentile	29	29	29	29
25th percentile	28	28	28	28
10th percentile	27	27	26	27
5th percentile	26	26	26	26
1st percentile	25	25	25	24
mean (SD)	28.9 (1.2)	28.7 (1.3)	28.6 (1.4)	28.5 (1.4)
Education > 13 years	n = 108	n = 59	n = 127	n = 91
99th percentile	30	30	30	30
95th percentile	30	30	30	30
90th percentile	30	30	30	30
75th percentile	30	30	30	30
50th percentile	29	30	29	29
25th percentile	29	29	28	28
10th percentile	27	27	28	27
5th percentile	27	26	27	26
1st percentile	26	25	26	25
mean (SD)	29.1 (1.1)	29.2 (1.3)	29.0 (1.0)	28.7 (1.3)
**MoCA**	Women		Men	
	70-74 years	≥ 75 years	70-74 years	≥ 75 years
Education ≤ 13 years	n = 217	n = 223	n = 88	n = 91
99th percentile	30	30	30	30
95th percentile	30	29	30	29
90th percentile	29	28	29	29
75th percentile	28	27	27	27
50th percentile	27	25	25.5	25
25th percentile	24	23	23	23
10th percentile	23	21	20	20
5th percentile	21	20	20	19
1st percentile	18	16	17	16
mean (SD)	26.3 (2.7)	25.0 (2.9)	25.0 (3.1)	24.8 (3.1)
Education > 13 years	n = 108	n = 59	n = 127	n = 91
99th percentile	30	30	30	30
95th percentile	30	30	30	30
90th percentile	30	30	29	29
75th percentile	29	29	29	28
50th percentile	28	27	27	26
25th percentile	26	24	25	24
10th percentile	24	23	23	21
5th percentile	23	22	23	20
1st percentile	21	18	19	17
mean (SD)	27.2 (2.3)	26.3 (2.8)	26.6 (2.4)	25.7 (2.9)

Supplemental Table 3. Percentiles for MMSE and MoCA scores by sex, age and country-specific education for Germany

**Germany (N = 350)**				
**MMSE**	Women		Men	
	70-74 years	≥ 75 years	70-74 years	≥ 75 years
Education ≤ 14 years	n = 115	n = 32	n = 27	n = 7
99th percentile	30	30	30	30
95th percentile	30	30	30	30
90th percentile	30	30	30	30
75th percentile	30	30	30	29
50th percentile	29	29	28	29
25th percentile	28	28.5	27	27
10th percentile	27	27	26	24
5th percentile	26	26	24	24
1st percentile	26	25	24	24
mean (SD)	28.6 (1.2)	28.8 (1.3)	28.2 (1.7)	28.0 (2.0)
Education > 14 years	n = 79	n = 21	n = 47	n = 22
99th percentile	30	30	30	30
95th percentile	30	30	30	30
90th percentile	30	30	30	29
75th percentile	30	30	30	29
50th percentile	29	29	29	29
25th percentile	28	29	28	28
10th percentile	27	28	28	27
5th percentile	26	28	27	27
1st percentile	24	25	27	26
mean (SD)	28.9 (1.4)	29.1 (1.2)	28.9 (1.0)	28.6 (1.0)
**MoCA**	Women		Men	
	70-74 years	≥ 75 years	70-74 years	≥ 75 years
Education ≤ 14 years	n = 115	n = 32	n = 27	n = 7
99th percentile	29	28	28	26
95th percentile	28	27	28	26
90th percentile	28	27	28	26
75th percentile	27	27	26	25
50th percentile	25	26	25	22
25th percentile	23	23	21	21
10th percentile	22	22	21	21
5th percentile	21	21	20	21
1st percentile	17	20	20	21
mean (SD)	25.0 (2.5)	24.9 (2.2)	24.2 (2.6)	22.9 (2.0)
Education > 14 years	n = 79	n = 21	n = 47	n = 22
99th percentile	30	29	29	28
95th percentile	29	29	28	27
90th percentile	29	28	27	27
75th percentile	28	28	27	25
50th percentile	26	26	25	24
25th percentile	24	24	23	22
10th percentile	23	23	22	22
5th percentile	22	23	22	20
1st percentile	19	21	20	19
mean (SD)	25.9 (2.2)	25.7 (2.2)	24.9 (2.0)	23.8 (2.2)

Supplemental Table 4. Percentiles for MMSE and MoCA scores by sex, age and country-specific education for Austria

**Austria (N = 199)**				
**MMSE**	Women		Men	
	70-74 years	≥ 75 years	70-74 years	≥ 75 years
Education ≤ 11 years	n = 44	n = 18	n = 26	n = 14
99th percentile	30	30	30	30
95th percentile	30	30	30	30
90th percentile	30	30	30	30
75th percentile	30	29	29	29
50th percentile	29	29	28.5	29
25th percentile	28	28	27	28
10th percentile	27	27	26	28
5th percentile	27	26	26	27
1st percentile	27	26	26	27
mean (SD)	28.9 (1.0)	28.6 (1.2)	28.4 (1.3)	28.6 (1.7)
Education > 11 years	n = 29	n = 11	n = 40	n = 17
99th percentile	30	30	30	30
95th percentile	30	30	30	30
90th percentile	30	30	30	30
75th percentile	30	30	30	30
50th percentile	30	28	29	29
25th percentile	29	28	28	28
10th percentile	28	28	27	27
5th percentile	28	26	27	24
1st percentile	27	26	26	24
mean (SD)	29.3 (0.9)	28.6 (1.3)	29.0 (1.1)	28.8 (0.9)
**MoCA**	Women		Men	
	70-74 years	≥ 75 years	70-74 years	≥ 75 years
Education ≤ 11 years	n = 44	n = 18	n = 26	n = 14
99th percentile	30	29	28	30
95th percentile	30	29	28	30
90th percentile	29	29	28	29
75th percentile	27	28	27	27
50th percentile	27	25.5	25	26
25th percentile	25	23	22	24
10th percentile	23	21	20	19
5th percentile	22	17	20	18
1st percentile	22	17	20	18
mean (SD)	26.6 (2.4)	25.3 (3.3)	24.6 (2.7)	25.3 (3.4)
Education > 11 years	n = 29	n = 11	n = 40	n = 17
99th percentile	30	30	30	30
95th percentile	30	30	30	30
90th percentile	30	29	30	29
75th percentile	30	27	28.5	27
50th percentile	28	26	27	27
25th percentile	25	24	25	25
10th percentile	23	23	24	24
5th percentile	22	22	23	22
1st percentile	22	22	23	22
mean (SD)	27.2 (2.6)	26.0 (2.4)	26.8 (2.1)	26.4 (2.0)

Supplemental Table 5. Percentiles for MMSE and MoCA scores by sex, age and country-specific education for France

France (N = 297)				
**MMSE**	Women		Men	
	70-74 years	≥ 75 years	70-74 years	≥ 75 years
Education ≤ 14 years	n = 51	n = 74	n = 28	n = 32
99th percentile	30	30	30	30
95th percentile	30	30	30	30
90th percentile	30	30	30	30
75th percentile	29	29	29.5	29
50th percentile	28	28	28.5	28
25th percentile	27	27	27	27
10th percentile	25	25	26	25
5th percentile	25	25	26	24
1st percentile	24	24	25	24
mean (SD)	28.0 (1.6)	27.7 (1.7)	28.3 (1.3)	28.0 (1.7)
Education > 14 years	n = 27	n = 27	n = 33	n = 25
99th percentile	30	30	30	30
95th percentile	30	30	30	30
90th percentile	30	30	30	30
75th percentile	30	29	29	29
50th percentile	29	29	29	29
25th percentile	28	28	28	28
10th percentile	26	27	27	27
5th percentile	26	27	25	26
1st percentile	26	27	25	25
mean (SD)	28.8 (1.3)	28.6 (1.0)	28.6 (1.4)	28.4 (1.3)
**MoCA**	Women		Men	
	70-74 years	≥ 75 years	70-74 years	≥ 75 years
Education ≤ 14 years	n = 51	n = 74	n = 28	n = 32
99th percentile	30	30	29	30
95th percentile	30	29	29	30
90th percentile	29	29	29	29
75th percentile	29	28	27	28
50th percentile	27	26	26	26
25th percentile	24	24	25	24
10th percentile	23	22	23	22
5th percentile	22	19	22	18
1st percentile	19	17	22	17
mean (SD)	26.3 (2.6)	25.7 (3.0)	26.0 (2.0)	25.7 (3.1)
Education > 14 years	n = 27	n = 27	n = 33	n = 25
99th percentile	30	30	30	30
95th percentile	30	30	30	30
90th percentile	30	30	30	29
75th percentile	29	29	29	28
50th percentile	28	27	28	27
25th percentile	26	26	27	26
10th percentile	24	25	25	25
5th percentile	22	24	24	24
1st percentile	22	23	23	23
mean (SD)	27.4 (2.1)	27.1 (1.9)	27.8 (1.9)	27.1 (1.7)

Supplemental Table 6. Percentiles for MMSE and MoCA scores by sex, age and country-specific education for Portugal

**Portugal (N = 301)**				
**MMSE**	Women		Men	
	70-74 years	≥ 75 years	70-74 years	≥ 75 years
Education ≤ 6 years	n = 57	n = 59	n = 17	n = 29
99th percentile	30	30	30	30
95th percentile	29	29	30	29
90th percentile	28	29	30	29
75th percentile	27	27	29	29
50th percentile	26	26	28	28
25th percentile	26	24	26	26
10th percentile	24	24	25	24
5th percentile	24	24	25	24
1st percentile	24	24	25	24
mean (SD)	26.3 (1.6)	26.2 (1.7)	27.5 (1.9)	27.2 (1.8)
Education > 6 years	n = 40	n = 36	n = 34	n = 29
99th percentile	30	30	30	30
95th percentile	30	30	30	30
90th percentile	30	30	30	30
75th percentile	30	29	30	29
50th percentile	28	28	29	28
25th percentile	27	26	27	27
10th percentile	26	25	26	25
5th percentile	26	24	26	24
1st percentile	26	24	25	24
mean (SD)	28.2 (1.5)	27.6 (1.8)	28.4 (1.5)	28.0 (1.7)
**MoCA**	Women		Men	
	70-74 years	≥ 75 years	70-74 years	≥ 75 years
Education ≤ 6 years	n = 57	n = 59	n = 17	n = 29
99th percentile	27	27	26	26
95th percentile	25	25	26	25
90th percentile	24	23	25	24
75th percentile	22	21	24	22
50th percentile	20	17	23	20
25th percentile	17	14	19	17
10th percentile	13	12	12	14
5th percentile	13	10	10	13
1st percentile	11	9	10	13
mean (SD)	19.5 (3.7)	17.1 (4.4)	20.7 (4.7)	19.6 (3.6)
Education > 6 years	n = 40	n = 36	n = 34	n = 29
99th percentile	30	28	30	30
95th percentile	29	28	28	28
90th percentile	28	27	27	28
75th percentile	27	26	26	26
50th percentile	25	23	24.5	24
25th percentile	22	21	22	20
10th percentile	18	19	21	19
5th percentile	16	18	20	18
1st percentile	16	16	20	17
mean (SD)	24.1 (3.7)	23.1 (3.2)	24.3 (2.4)	23.6 (3.6)

Supplemental Table 7. Percentiles for MMSE and MoCA scores by sex, age and education (cutoff 12 years) for Switzerland

**Switzerland (N = 1004)**				
**MMSE**	Women		Men	
	70-74 years	≥ 75 years	70-74 years	≥ 75 years
Education ≤ 12 years	n = 179	n = 195	n = 52	n = 59
99th percentile	30	30	30	30
95th percentile	30	30	30	30
90th percentile	30	30	30	30
75th percentile	30	30	30	30
50th percentile	29	29	29	29
25th percentile	28	28	28	28
10th percentile	27	27	27	26
5th percentile	26	26	25	26
1st percentile	25	24	25	24
mean (SD)	28.8 (1.3)	28.7 (1.3)	28.6 (1.4)	28.7 (1.4)
Education > 12 years	n = 146	n = 87	n = 163	n = 123
99th percentile	30	30	30	30
95th percentile	30	30	30	30
90th percentile	30	30	30	30
75th percentile	30	30	30	30
50th percentile	29	30	29	29
25th percentile	29	29	28	28
10th percentile	28	27	27	27
5th percentile	27	27	27	26
1st percentile	26	25	26	25
mean (SD)	29.1 (1.0)	29.1 (1.3)	28.9 (1.1)	28.6 (1.3)
**MoCA**	Women		Men	
	70-74 years	≥ 75 years	70-74 years	≥ 75 years
Education ≤ 12 years	n = 179	n = 195	n = 52	n = 59
99th percentile	30	30	30	30
95th percentile	30	29	29	29
90th percentile	29	28	29	28
75th percentile	28	27	27	26
50th percentile	27	25	25.5	25
25th percentile	24	23	23	22
10th percentile	23	21	22	20
5th percentile	21	20	18	19
1st percentile	18	15	17	16
mean (SD)	26.2 (2.7)	24.9 (2.8)	25.0 (3.1)	24.4 (3.1)
Education > 12 years	n = 146	n = 87	n = 163	n = 123
99th percentile	30	30	30	30
95th percentile	30	30	30	30
90th percentile	30	29	29	29
75th percentile	29	29	28	28
50th percentile	28	26	27	26
25th percentile	26	24	24	23
10th percentile	24	23	23	21
5th percentile	23	21	21	20
1st percentile	21	16	19	19
mean (SD)	27.2 (2.4)	26.0 (3.0)	26.3 (2.7)	25.6 (3.0)

Supplemental Table 8. Percentiles for MMSE and MoCA scores by sex, age and education (cutoff 12 years) for Germany

**Germany (N = 350)**				
**MMSE**	Women		Men	
	70-74 years	≥ 75 years	70-74 years	≥ 75 years
Education ≤ 12 years	n = 67	n = 19	n = 15	n = 5
99th percentile	30	30	30	30
95th percentile	30	30	30	30
90th percentile	30	30	30	30
75th percentile	29	30	30	29
50th percentile	29	29	28	29
25th percentile	28	29	28	27
10th percentile	26	27	27	24
5th percentile	26	26	24	24
1st percentile	24	26	24	24
mean (SD)	28.5 (1.4)	29.9 (1.2)	28.4 (1.6)	27.8 (2.4)
Education > 12 years	n = 127	n = 34	n = 59	n = 24
99th percentile	30	30	30	30
95th percentile	30	30	30	30
90th percentile	30	30	30	29
75th percentile	30	30	30	29
50th percentile	29	29	29	29
25th percentile	28	29	28	28
10th percentile	27	28	27	27
5th percentile	27	25	27	27
1st percentile	24	25	24	26
mean (SD)	28.9 (1.2)	29.0 (1.3)	28.7 (1.2)	28.6 (0.9)
**MoCA**	Women		Men	
	70-74 years	≥ 75 years	70-74 years	≥ 75 years
Education ≤ 12 years	n = 67	n = 19	n = 15	n = 5
99th percentile	29	27	28	25
95th percentile	28	27	28	25
90th percentile	28	27	28	25
75th percentile	27	27	26	22
50th percentile	25	26	24	22
25th percentile	23	23	21	21
10th percentile	21	21	21	21
5th percentile	20	21	20	21
1st percentile	17	21	20	21
mean (SD)	24.7 (2.6)	24.8 (2.1)	23.8 (2.5)	22.2 (1.6)
Education > 12 years	n = 127	n = 34	n = 59	n = 24
99th percentile	29	29	29	28
95th percentile	29	29	28	27
90th percentile	28	28	27	27
75th percentile	27	27	27	25.5
50th percentile	26	26	25	24
25th percentile	24	24	23	22
10th percentile	23	23	22	22
5th percentile	22	21	21	20
1st percentile	20	20	20	19
mean (SD)	25.7 (2.2)	25.4 (2.3)	24.9 (2.2)	23.9 (2.2)

Supplemental Table 9. Percentiles for MMSE and MoCA scores by sex, age and education (cutoff 12 years) for Austria

**Austria (N = 199)**				
**MMSE**	Women		Men	
	70-74 years	≥ 75 years	70-74 years	≥ 75 years
Education ≤ 12 years	n = 58	n = 25	n = 40	n = 21
99th percentile	30	30	30	30
95th percentile	30	30	30	30
90th percentile	30	30	30	30
75th percentile	30	30	30	30
50th percentile	29	29	29	29
25th percentile	28	28	28	28
10th percentile	28	27	27	27
5th percentile	27	26	26	27
1st percentile	27	26	26	24
mean (SD)	29.1 (1.0)	28.6 (1.3)	28.6 (1.2)	28.6 (1.5)
Education > 12 years	n = 15	n = 4	n = 26	n = 10
99th percentile	30	30	30	30
95th percentile	30	30	30	30
90th percentile	30	30	30	30
75th percentile	30	29	30	29
50th percentile	29	28	30	29
25th percentile	28	28	28	28
10th percentile	28	28	27	28
5th percentile	27	28	27	28
1st percentile	27	28	26	28
mean (SD)	29.1 (1.0)	28.5 (1.0)	29.1 (1.2)	28.8 (0.8)
**MoCA**	Women		Men	
	70-74 years	≥ 75 years	70-74 years	≥ 75 years
Education ≤ 12 years	n = 58	n = 25	n = 40	n = 21
99th percentile	30	29	30	30
95th percentile	30	29	28.5	30
90th percentile	30	29	28	29
75th percentile	29	27	27	27
50th percentile	27	26	25.5	26
25th percentile	25	24	23.5	25
10th percentile	23	22	21.5	23
5th percentile	22	21	20	19
1st percentile	22	17	20	18
mean (SD)	26.7 (2.4)	25.4 (3.0)	25.2 (2.5)	25.8 (3.0)
Education > 12 years	n = 15	n = 4	n = 26	n = 10
99th percentile	30	30	30	29
95th percentile	30	30	30	29
90th percentile	30	30	30	28.5
75th percentile	30	29.5	29	27
50th percentile	29	27	28	26.5
25th percentile	26	24	25	25
10th percentile	22	23	24	23
5th percentile	22	23	23	22
1st percentile	22	23	23	22
mean (SD)	27.6 (2.9)	26.8 (3.3)	27.2 (2.3)	26.0 (2.1)

Supplemental Table 10. Percentiles for MMSE and MoCA scores by sex, age and education (cutoff 12 years) for France

France (N = 297)				
**MMSE**	Women		Men	
	70-74 years	≥ 75 years	70-74 years	≥ 75 years
Education ≤ 12 years	n = 32	n = 56	n = 21	n = 23
99th percentile	30	30	30	30
95th percentile	30	30	30	30
90th percentile	30	30	30	30
75th percentile	29	29	29	29
50th percentile	28	27.5	28	28
25th percentile	27	26	27	27
10th percentile	26	25	26	25
5th percentile	25	24	26	24
1st percentile	25	24	25	24
mean (SD)	26.4 (2.4)	25.3 (3.0)	28.1 (1.5)	27.6 (1.8)
Education > 12 years	n = 46	n = 45	n = 40	n = 34
99th percentile	30	30	30	30
95th percentile	30	30	30	30
90th percentile	30	30	30	30
75th percentile	30	29	30	29
50th percentile	29	29	29	29
25th percentile	28	28	28	28
10th percentile	26	27	27	27
5th percentile	25	27	25	26
1st percentile	24	25	25	25
mean (SD)	26.9 (2.5)	27.0 (2.2)	28.6 (1.4)	28.5 (1.2)
**MoCA**	Women		Men	
	70-74 years	≥ 75 years	70-74 years	≥ 75 years
Education ≤ 12 years	n = 32	n = 56	n = 21	n = 23
99th percentile	30	30	29	30
95th percentile	30	29	29	29
90th percentile	29	29	29	28
75th percentile	28.5	27.5	27	27
50th percentile	26.5	26	26	25
25th percentile	24.5	24	25	23
10th percentile	23	21	23	21
5th percentile	23	18	22	18
1st percentile	21	17	22	17
mean (SD)	28.1 (1.4)	27.5 (1.7)	25.7 (2.0)	24.9 (3.2)
Education > 12 years	n = 46	n = 45	n = 40	n = 34
99th percentile	30	30	30	30
95th percentile	30	30	30	30
90th percentile	30	29	30	29
75th percentile	29	29	29	28
50th percentile	27	28	28	27
25th percentile	26	26	27	26
10th percentile	23	4	25	25
5th percentile	22	23	24	24
1st percentile	19	21	23	23
mean (SD)	28.4 (1.7)	28.5 (1.2)	27.7 (1.9)	27.3 (1.6)

Supplemental Table 11. Percentiles for MMSE and MoCA scores by sex, age and education (cutoff 12 years) for Portugal

**Portugal (N = 301)**				
**MMSE**	Women		Men	
	70-74 years	≥ 75 years	70-74 years	≥ 75 years
Education ≤ 12 years	n = 77	n = 80	n = 35	n = 46
99th percentile	30	30	30	30
95th percentile	30	30	30	30
90th percentile	29	29	30	29
75th percentile	28	28	29	29
50th percentile	27	27	28	28
25th percentile	26	26	26	26
10th percentile	24	24	25	24
5th percentile	24	24	25	24
1st percentile	24	24	25	24
mean (SD)	26.6 (1.7)	26.4 (1.8)	27.8 (1.8)	27.4 (1.9)
Education > 12 years	n = 20	n = 15	n = 16	n = 12
99th percentile	30	30	30	30
95th percentile	30	30	30	30
90th percentile	30	30	30	30
75th percentile	29	30	30	29
50th percentile	29	28	29	29
25th percentile	27.5	28	28	28.5
10th percentile	27	27	27	27
5th percentile	26.5	26	26	26
1st percentile	26	26	26	26
mean (SD)	28.7 (1.3)	28.7 (1.3)	28.8 (1.2)	28.7 (1.2)
**MoCA**	Women		Men	
	70-74 years	≥ 75 years	70-74 years	≥ 75 years
Education ≤ 12 years	n = 77	n = 80	n = 35	n = 46
99th percentile	29	27	28	25
95th percentile	28	27	28	25
90th percentile	28	27	28	25
75th percentile	27	27	26	22
50th percentile	25	26	24	22
25th percentile	23	23	21	21
10th percentile	21	21	21	21
5th percentile	20	21	20	21
1st percentile	17	21	20	21
mean (SD)	20.2 (4.1)	18.5 (4.8)	22.2 (3.8)	20.5 (3.6)
Education > 12 years	n = 20	n = 15	n = 16	n = 12
99th percentile	29	29	29	28
95th percentile	29	29	28	27
90th percentile	28	28	27	27
75th percentile	27	27	27	25.5
50th percentile	26	26	25	24
25th percentile	24	24	23	22
10th percentile	23	23	22	22
5th percentile	22	21	21	20
1st percentile	20	20	20	19
mean (SD)	25.8 (2.0)	23.9 (2.9)	25.1 (2.7)	26.0 (2.9)
